# Endothelial and inflammatory pathophysiology in dengue shock: New insights from a prospective cohort study in Vietnam

**DOI:** 10.1371/journal.pntd.0012071

**Published:** 2024-03-27

**Authors:** Angela McBride, Huynh Thi Le Duyen, Nguyen Lam Vuong, Phan Vinh Tho, Luong Thi Hue Tai, Nguyen Thanh Phong, Nguyen Thanh Ngoc, Lam Minh Yen, Phung Tran Huy Nhat, Tran Thuy Vi, Martin J. Llewelyn, Louise Thwaites, Nguyen Van Hao, Sophie Yacoub

**Affiliations:** 1 Oxford University Clinical Research Unit, Ho Chi Minh City, Vietnam; 2 Brighton and Sussex Medical School, Brighton, United Kingdom; 3 Nuffield Department of Medicine, University of Oxford, Oxford, United Kingdom; 4 University of Medicine and Pharmacy, Ho Chi Minh City, Vietnam; 5 Hospital for Tropical Disease, Ho Chi Minh City, Vietnam; University of Wisconsin-Madison, UNITED STATES

## Abstract

Dengue shock (DS) is the most severe complication of dengue infection; endothelial hyperpermeability leads to profound plasma leakage, hypovolaemia and extravascular fluid accumulation. At present, the only treatment is supportive with intravenous fluid, but targeted endothelial stabilising therapies and host immune modulators are needed. With the aim of prioritising potential therapeutics, we conducted a prospective observational study of adults (≥16 years) with DS in Vietnam from 2019–2022, comparing the pathophysiology underlying circulatory failure with patients with septic shock (SS), and investigating the association of biomarkers with clinical severity (SOFA score, ICU admission, mortality) and pulmonary vascular leak (daily lung ultrasound for interstitial and pleural fluid). Plasma was collected at enrolment, 48 hours later and hospital discharge. We measured biomarkers of inflammation (IL-6, ferritin), endothelial activation (Ang-1, Ang-2, sTie-2, VCAM-1) and endothelial glycocalyx breakdown (hyaluronan, heparan sulfate, endocan, syndecan-1). We enrolled 135 patients with DS (median age 26, median SOFA score 7, 34 required ICU admission, 5 deaths), together with 37 patients with SS and 25 healthy controls. Within the DS group, IL-6 and ferritin were associated with admission SOFA score (IL-6: βeta0.70, p<0.001 & ferritin: βeta0.45, p<0.001), ICU admission (IL-6: OR 2.6, p<0.001 & ferritin: OR 1.55, p<0.001) and mortality (IL-6: OR 4.49, p = 0.005 & ferritin: OR 13.8, p = 0.02); both biomarkers discriminated survivors and non-survivors at 48 hours and all patients who died from DS had pre-mortem ferritin ≥100,000ng/ml. IL-6 most strongly correlated with severity of pulmonary vascular leakage (R = 0.41, p<0.001). Ang-2 correlated with pulmonary vascular leak (R = 0.33, p<0.001) and associated with SOFA score (β 0.81, p<0.001) and mortality (OR 8.06, p = 0.002). Ang-1 was associated with ICU admission (OR 1.6, p = 0.005) and mortality (OR 3.62, p = 0.006). All 4 glycocalyx biomarkers were positively associated with SOFA score, but only syndecan-1 was associated with ICU admission (OR 2.02, p<0.001) and mortality (OR 6.51, p<0.001). This study highlights the central role of hyperinflammation in determining outcomes from DS; the data suggest that anti-IL-1 and anti-IL-6 immune modulators and Tie2 agonists may be considered as candidates for therapeutic trials in severe dengue.

## Author summary

Dengue is the most common arboviral infection worldwide. Dengue shock is the most severe complication of dengue infection; patients develop marked plasma leakage, hypovolaemia and extravascular fluid accumulation. At present, the only treatment available is intravenous fluid, but targeted therapies are needed. With the aim of prioritizing potential therapeutics, we conducted a prospective observational study of 135 adults with dengue shock in Vietnam, investigating the association between biomarkers of inflammation, endothelial activation and endothelial glycocalyx breakdown and clinical severity, and comparing pathophysiology with patients with septic shock and healthy controls. In the dengue shock group, 5/135 patients died. The inflammatory biomarkers IL-6 and ferritin were associated with SOFA score, requirement for ICU admission and mortality. IL-6 most strongly correlated with severity of vascular leakage in the lung, measured by ultrasound. The endothelial biomarkers Angiopoietin-2 and syndecan-1 were both also associated with SOFA score and mortality. Our findings highlight the central role of hyperinflammation in determining outcomes from dengue shock; the data suggest that anti-IL-1 and anti-IL-6 immune modulators and Tie2 agonists may be considered as candidates for therapeutic trials in severe dengue.

## Introduction

Dengue is the most abundant arboviral infection worldwide, causing an estimated 96 million symptomatic infections each year [1]. With urbanization and climate change, the population at risk of dengue is constantly expanding. In most cases, dengue infection is non-severe and self-limiting; defervescence occurs when the host immune response clears the viraemia, usually between days 4–6 of illness. However, coincident with the host immune response, a minority of patients develop severe dengue, usually manifest as severe plasma leakage leading to hypovolaemic shock, haemorrhage, and/or organ impairment. The risk of severe dengue is highest during secondary infection; partially primed but ineffective humoral and cell-mediated immune responses are thought to amplify viral load, lead to unchecked macrophage activation, and drive excessive proinflammatory cytokine-mediated tissue damage [2,3].

Although there have been promising recent developments with Wolbachia biocontrol and licensing of a tetravalent vaccine for dengue, there are no treatments known to alter the course of established disease: no antivirals or host-directed therapeutics have shown efficacy to date. As a neglected tropical disease, few therapeutics have been developed with dengue specifically in mind; the preclinical drug development pipeline has been impeded further by the absence of an adequate animal model of plasma leakage to support screening for early phase clinical trials. However, considering the underlying pathophysiology, two main strategies emerge as potential targets for host-directed treatments: 1) modulation of the host immune response driving endothelial (and other tissue) damage and 2) attenuating endothelial cell activation/glycocalyx breakdown to reduce plasma leakage. Strategies to dampen the host immune response to severe infection have saved lives from COVID-19, and shown real promise in sepsis/septic shock [4–6]. There are no systemic anti-permeability therapeutics currently in clinical use, but some of the pathways implicated in plasma leakage do have drugs in clinical trials for other severe infections (e.g. the Tie2 agonist AV001, Vasomune Therapeutics, ClinicalTrials.gov: NCT05123755).

The aim of this study was to compare the inflammatory and endothelial pathophysiology underlying microvascular leak in dengue shock and septic shock, with the aim that such data may provide an evidence base to accelerate the translation of promising endothelial and immune therapeutics from sepsis to dengue, clarify time windows within which therapeutic agents may be useful, and prioritise specific pathways for the development of novel therapeutics. With respect to dengue shock, we also aimed to investigate the association of biomarkers of inflammation, endothelial activation and glycocalyx breakdown with clinical outcomes including Sequential Organ Failure Assessment (SOFA) score, ICU admission and mortality.

## Methods

### Ethics statement

Ethical approval was obtained from the Oxford Tropical Research Ethics Committee and the Ethics review Committee at HTD. Written informed consent was obtained from all participants, or their representative if they did not have capacity. For participants aged 16–17 years, a parent/guardian provided written consent, with additional written assent provided by the participant.

### Clinical study

We conducted a prospective observational cohort study of patients aged ≥16 years with dengue shock, admitted to the Hospital for Tropical Diseases (HTD) in Ho Chi Minh City (HCMC), Vietnam between 2019 and 2022. Comparator groups included patients with septic shock and healthy controls.

Patients with dengue shock and septic shock were recruited within 24 hours of the diagnosis of shock. Participants transferred to HTD from other hospitals were eligible for recruitment, provided ≤24 hours had elapsed between diagnosis and first research assessments. Diagnostic criteria for dengue shock and septic shock were in line with the WHO definition (2009) and Sepsis 3 definitions, respectively [7,8]. We excluded patients who were pregnant, patients with dengue shock admitted on ≥day 7 of illness, and patients who developed septic shock ≥48 hours after admission to hospital.

After informed consent, all patients underwent detailed assessments of disease severity, organ support requirements, treatments received, and lung ultrasound; assessments continued daily until discharge or for up to 7 days, whichever sooner. At study enrolment, 48 hours later, and at hospital discharge, plasma samples were collected for endothelial and inflammatory biomarkers, and the reactive hyperaemia index (RHI) was measured with the EndoPAT device. Participants received standard supportive care. All clinical and laboratory assessments were entered into a structured Case Report Form.

Since population normative data is not available in Vietnam for either the biomarkers or RHI, 25 frequency age-matched healthy controls were recruited from within the staff network at the Oxford University Clinical Research Unit, HCMC. Controls were eligible if they did not report any of the following: chronic illness, regular medications, current pregnancy, any febrile illness in the prior 28 days, or diagnosis of/treatment for dengue or bacterial infection in the previous 6 months. Healthy control participants underwent phlebotomy and EndoPAT assessment at a single time-point.

### Lung ultrasound

All patients underwent daily lung ultrasound, performed using a standardised operating procedure based on the Kigali ARDS protocol [9]: assessment for B-lines, consolidation and pleural effusion was performed at 6-points on each side of the chest (2 anterior, 2 lateral, 2 posterolateral), and the worst pattern seen in each zone scored as follows: A lines only = 0, B1 pattern = 1, B2 pattern = 2, Consolidation/collapse = 3, Effusion = 4. The ‘pulmonary vascular leak score’, developed for this study, was calculated as the sum of all 12 zone scores (score range 0–48). If present, the depth of pleural effusion was measured at both lung bases; the pleural ‘effusion score’ was calculated as the sum of both depth measurements in centimeters.

### Endothelial function testing

Endothelial nitric oxide dependent vasodilatory function was tested by peripheral artery tonometry using the FDA cleared EndoPAT device, whereby digital pulse volume changes during reactive hyperaemia are measured using pneumatic finger probes as per standardised methods [10]. Due to the preceding arterial occlusion requiring inflation of the blood pressure cuff to ≥200mmHg, the assessment was omitted in patients with platelet count <20x10^9^/L or disseminated intravascular coagulation (DIC) in order to avoid excessive bruising.

### Laboratory methods

#### Virological and microbiological diagnostics

Plasma from patients diagnosed clinically with dengue underwent serotype specific quantitative real time reverse transcriptase PCR using an in-house assay described elsewhere [11]. Patients with negative PCR were not excluded from analysis, since the onset of dengue shock often coincides with viral clearance from the blood, and patients were treated as having dengue shock. For patients with septic shock, routine microbiological investigations were performed in line with hospital guidelines and the patient’s presenting syndrome. Where the causative organism was confirmed, culture results were recorded, otherwise the presumed source of sepsis was recorded at hospital discharge.

#### Inflammatory and endothelial biomarkers

Plasma was frozen within 2 hours of collection, and later tested using commercial assays for inflammatory and endothelial biomarkers. Ferritin and IL-6 were measured using the Elecsys electrochemiluminescence immunoassay on the Cobas e 411 analyzer (Roche system). The following biomarkers were analysed using a multiplexed magnetic bead assay on a Luminex 200 platform according to the manufacturer’s specifications: Angiopoietin-1 (Ang-1), Angiopoietin-2 (Ang-2), soluble Tie-2, Vascular Cell Adhesion Molecule 1 (VCAM-1), Endocan and atrial natriuretic peptide (ANP) (R&D systems). The following biomarkers were analysed by ELISA: hyaluronan (Cat. No: DHYAL, R&D systems), heparan sulfate (Cat. No: abx513537; Abbexa) and syndecan-1 (SDC-1, Cat. No: 950.640.91; Diaclone), in accordance with the manufacturer’s specifications.

### Statistical analysis

Descriptive statistics were used to summarise the characteristics of the patient cohort and draw comparisons between groups with dengue shock and septic shock, using proportions for categorical data and median (25^th^;75^th^ centiles) for continuous data.

Due to skewed distribution, the biomarkers have been log-2 transformed for analysis and graphical presentation. However, the inflammatory biomarkers ferritin and IL-6 have been reported in untransformed form, to allow clinical interpretation. Wilcoxon-signed rank test was used to compare biomarkers between patients with dengue shock, septic shock and healthy controls. Since this was an exploratory analysis only, no correction was made for multiple comparisons, but acknowledging this limitation, a p value of <0.01 was selected to signify statistically significant differences.

The association between biomarkers and SOFA score was investigated using linear regression, whereas logistic regression was used to investigate the association of biomarkers with the categorical endpoints: ICU admission and mortality. Due to limited sample size, no additional covariates were included in the models. Relationships between markers of extravascular fluid leak measured by lung ultrasound and biomarkers were assessed by the Spearman’s correlation coefficient. Statistical analyses were conducted in Stata version 17.0 and R statistical software version 4.1.0.

## Results

### Baseline characteristics and outcomes

Table 1 summarises the baseline demographics, clinical characteristics and in-hospital outcomes of patients with dengue shock (DS, n = 135) and septic shock (SS, n = 35). Results of diagnostic testing are reported in Table 2. Patients with DS were younger (median age 25 for DS versus 55 for SS, p<0.001), less likely to be smokers, had fewer comorbidities, and less likely to be taking regular medication than those with SS. By design, the healthy controls had no known comorbidities, and took no regular medication. Since the healthy control group was frequency age matched to the whole cohort, the distribution of healthy controls was more similar to the DS group than the SS group, as the former was substantially larger; the median (25^th^;75^th^ centile) age was 29 (24; 39) with range 17–67 years. There were more females in the control group (21/25 (84%)).

**Table 1:** Baseline demographic, clinical characteristics and clinical outcomes.

	n	Dengue shock (N = 135)^1^	n	Septic shock (N = 37)^1^	p-value^2^
**Demographics**					
Age, years	135	26 (20; 33)	37	55 (47; 63)	**<0.001**
Sex male	135	66 (49)	37	23 (62)	0.194
Smoker	135	0 (0)	37	8 (22)	**<0.001**
BMI (kg/m2)	135	22.9 (20.3; 26.3)	37	23.2 (20.8; 25.8)	0.788
BMI category (Asian cut-offs)	135		37		0.747
Acceptable		68 (50)		18 (49)	
Overweight		43 (32)		14 (38)	
Obese		24 (18)		5 (14)	
Charlson comorbidity index	135		37		**<0.001**
0		122 (90)		10 (27)	
1		10 (7)		7 (19)	
2		0 (0)		5 (14)	
3		3 (2)		4 (11)	
4		0 (0)		2 (5)	
5		0 (0)		4 (11)	
6		0 (0)		4 (11)	
7		0 (0)		1 (3)	
Taking regular medication	135	2 (1)	37	14 (38)	
**Baseline clinical characteristics**					
Day of illness at enrolment*	135	5 (4; 6)	37	3 (2; 5)	**<0.001**
Transferred from another hospital	135	80 (59)	37	13 (35)	0.015
Received antibiotics prior to enrolment	132	2 (2)	37	10 (27)	**<0.001**
Received IV fluid prior to enrolment	134	64 (48)	37	12 (32)	0.134
Volume of pre-enrolment IV fluid (ml)	59	2000 (1500; 3000)	12	1500 (564; 2151)	0.122
Lowest mean arterial pressure in first 24 hours	134	80 (73; 86)	37	52 (50; 60)	**<0.001**
Maximum heart rate in first 24 hours	135	102 (90; 115)	37	120 (112; 140)	**<0.001**
SOFA score at enrolment	135	7 (6; 7)	37	10 (9; 13)	**<0.001**
**Clinical outcomes**					
In-hospital death	135	5 (4)	37	11 (30)	**<0.001**
ICU admission	135	34 (25)	37	30 (81)	**<0.001**
Days in ICU (if admitted)	34	4 (2; 5)	30	7 (4; 10)	0.010
Hospital length of stay (days)	135	5 (4; 7)	37	11 (7; 15)	**<0.001**
Required invasive/non-invasive ventilation	135	13 (10)	37	19 (51)	**<0.001**
Required invasive ventilation	135	9 (7)	37	16 (43)	**<0.001**
Duration of invasive ventilation (days)	9	7 (4; 8)	16	5 (3; 7)	0.332
Duration of non-invasive ventilation (days)	7	1 (1; 5)	8	6 (4; 6)	0.206
Required vasopressors	135	8 (6)	37	35 (95)	**<0.001**
Duration of vasopressors (days)	8	3 (2; 5)	35	3 (2; 4)	0.750
Duration of IV fluid requirement	135	2 (2; 2)	37	4 (2; 7)	**<0.001**
Total volume of IV fluid received (ml)	135	4830 (3589; 6455)	37	5165 (3500; 8505)	0.344
Required haemofiltration	135	7 (5)	37	10 (27)	**<0.001**
Duration of haemofiltration (days)	7	4 (4; 7)	10	4 (2; 5)	0.485
Required transfusion of platelets^	135	20 (15)	37	0 (0)	**<0.001**
Required transfusion of fresh frozen plasma^	135	12 (9)	37	0 (0)	**<0.001**
Required transfusion of packed red cells^	135	17 (13)	37	0 (0)	**<0.001**
Maximum haematocrit (%)	135	53 (48; 56)	37	38 (35; 43)	**<0.001**
Day of max HCT (day of illness)	135	5 (4; 6)	36	5 (3; 6)	0.130
Developed dengue re-shock	135	28 (21)	10	0 (0)	NA

^*1*^ *Median (25%; 75%); n (%)*

^*2*^ *Wilcoxon rank sum test; Fishers exact test*

*illness day 1 is defined as the first day of fever

^4 patients each received 1 pool of platelets and 1 patient received fresh frozen plasma within the first 48 hours of hospital admission. All remaining blood products were transfused after the 48hour plasma sample was collected.

**Table 2:** Virological and microbiological diagnostic tests for dengue shock and septic shock.

**Diagnostics for dengue shock**	**n**	**Dengue shock** **(N=135)** ^**1**^
NS1 antigen performed (during routine clinical care)	135	65 (48)
NS1 antigen positive	65	60 (92)
Dengue RT-PCR positive	135	102 (76)
Dengue serotype	102	
1		20 (20)
2		79 (77)
3		0 (0)
4		3 (3)
**Diagnostics for septic shock**	**n**	**Septic shock** **(N=37)** ^**1**^
**Microbiological confirmation of causal pathogen**	37	17 (46)
Source of positive culture	16	
Blood		11 (69)
Sputum		1 (6)
Skin/wound/blister swab		3 (19)
Ascites		1 (6)
**Bacteria isolated**	17	
**Gram negative**	10	
Acinetobacter baumanii		1
Burkholderia pseudomallei		1
Escherichia coli		1
Klebsiella pneumoniae		4
Vibrio vulnificus		1
Gram negative, not identified		2
**Gram positive**	6	
Staphylococcus aureus		1
Streptococcus anginosus		1
Streptococcus dysgalactiae		1
Streptococcus pyogenes		1
Gram positive cocci, not identified		2
**Other**	1	
Leptospira		1
**Presumed source of sepsis (if no positive culture)**	20	
Respiratory		10 (50)
Central nervous system		0 (0)
Skin/soft tissue		3 (15)
Abdominal		3 (15)
Urinary tract		3 (15)
Bone/joint		0 (0)
Unknown source		1 (5)

^1^n (%)

Patients with DS were enrolled on median (25^th^;75^th^ centile) illness day 5 (4;6), whereas patients with SS had been unwell for a shorter period (median illness day 3 (2;5)). A higher proportion of patients with DS had been transferred to HTD from another hospital compared to those with SS (59% for DS vs 35% for SS). Most patients who were transferred received intravenous fluid resuscitation prior to and/or during transfer.

**Fig 1:**
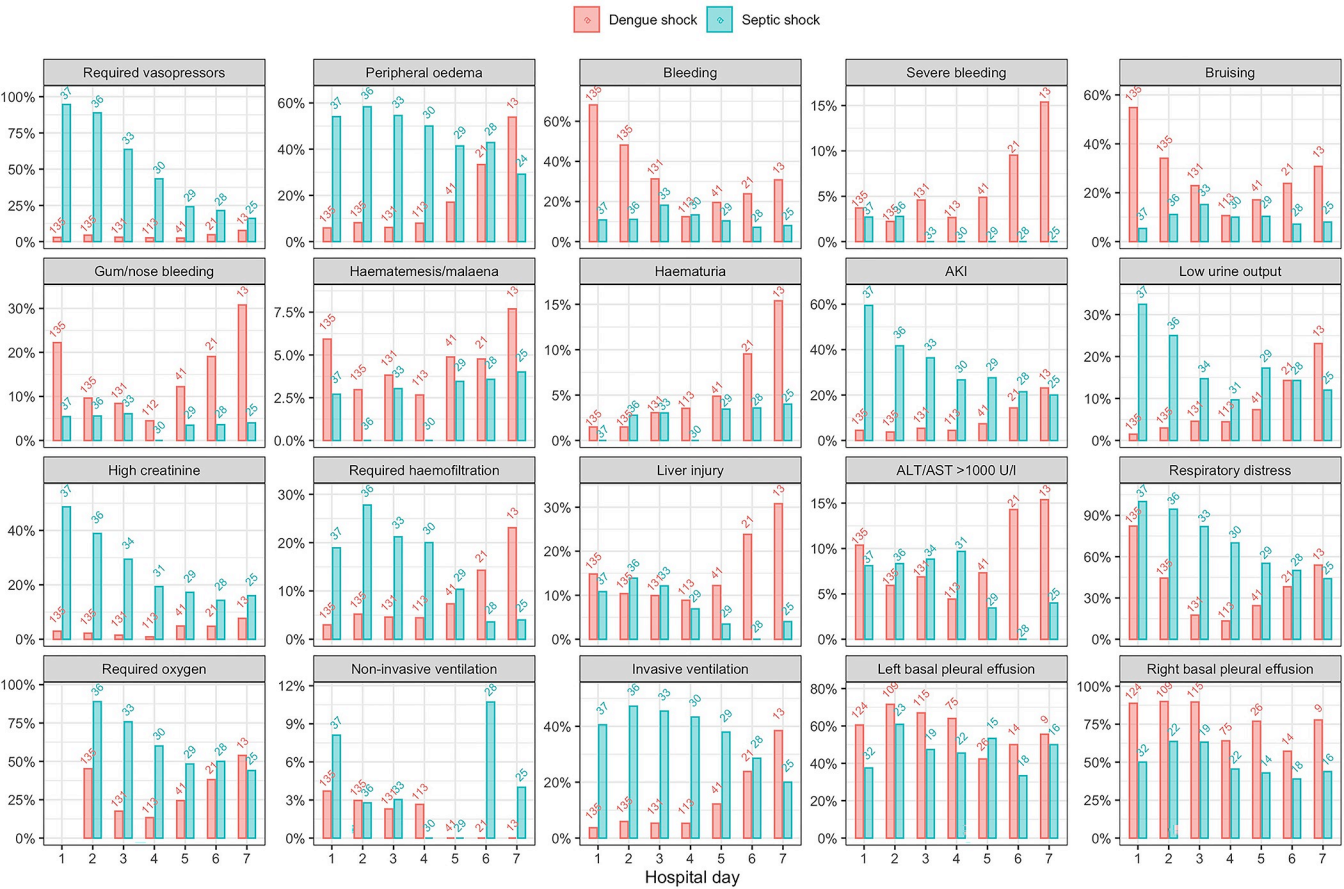
Clinical signs, endpoints and organ support interventions required for patients with dengue shock and septic shock. The numbers above the bars are the number of patients who were assessed for the corresponding outcomes on each day. Hospital day 1 = enrolment.

At enrolment, SOFA score was lower in the DS group versus the SS group (median SOFA 7 for DS versus 10 for SS, p<0.001). A higher proportion of patients with SS were admitted to ICU than patients with DS (81% for SS versus 25% for DS, p<0.001); patients can receive management for shock including IV fluids and vasopressors, but not other organ support, for up to 48 hours on the Emergency ward. The SS group had a higher proportion requiring ventilation, vasopressors, haemofiltration and required IV fluids for longer than the DS group (p<0.001 for all comparisons). 28/135 (21%) of DS patients developed re-shock, whereby an initial improvement in haemodynamic status after volume resuscitation was followed by a further episode of shock, indicating ongoing loss of circulatory volume by plasma leakage. Patients with SS stayed in ICU and hospital for longer than those with DS (p<0.001 for both comparisons). Fig 1 shows the clinical signs, organ failure and organ support requirements for all patients. Figs A and B in S1 Text display the results of vital signs, and initial haematological and biochemical laboratory parameters for patients with DS and SS.

Five patients (4%) with DS either died during hospital admission or were discharged to die at home. Eleven patients (30%) with SS either died in hospital or were discharged to die at home; significantly higher than the mortality rate for DS (p<0.001).

No patient with septic shock received blood products. Among the cohort with dengue shock, 20 patients received platelet transfusion (total 44 pools of platelets), 12 patients received fresh frozen plasma (total 93 units) and 17 patients received packed red cells (total 48 units). The majority of these blood products were administered after the 48 hour biomarker samples were taken; 4 patients each received 1 pool of platelets, and 1 patient received fresh frozen plasma within the first 48 hours of admission.

### Lung ultrasound

**Fig 2:**
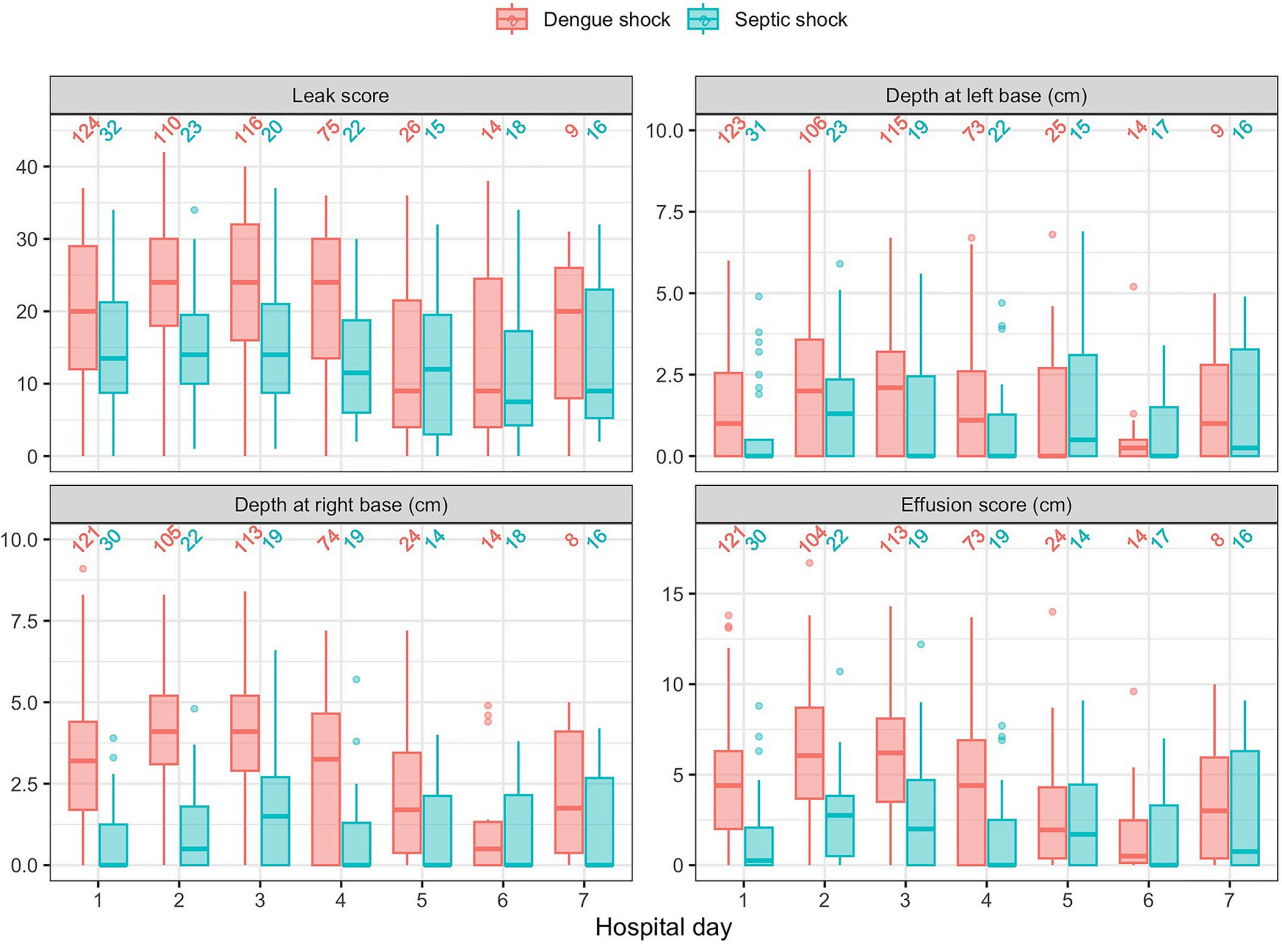
Serial pulmonary vascular leak score, depth of pleural effusion and effusion scores in participants with dengue shock and septic shock. The numbers above the bars are the number of patients who were assessed by lung ultrasound on each day. Hospital day 1 = enrolment.

The lung ultrasound analysis included data from 620 ultrasound scans (474 DS, 146 SS), with a total of 7440 individual lung windows, plus 1197 assessments for basal pleural effusion. Fig 2 shows the serial pulmonary vascular leak scores, depth of basal effusions, and pleural effusion scores. Although basal pleural effusion was common in both conditions, a higher proportion of DS patients had bi-basal pleural effusions (Fig 1). The pulmonary vascular leak score (reflecting both interstitial fluid and pleural effusion) and effusion score (combined depth of pulmonary effusions at both lung bases, cm) were higher in the DS group compared to the SS group until day 4. The depth of effusion at the right lung base was greater in patients with DS (Fig 2). Despite evidence of increased fluid in both the pulmonary interstitium and pleural spaces of DS patients, all clinical endpoints indicating respiratory compromise (respiratory distress, requirement for supplemental oxygen, non-invasive ventilation, invasive ventilation) occurred more frequently in the SS group (Fig 1).

### EndoPAT

**Fig 3:**
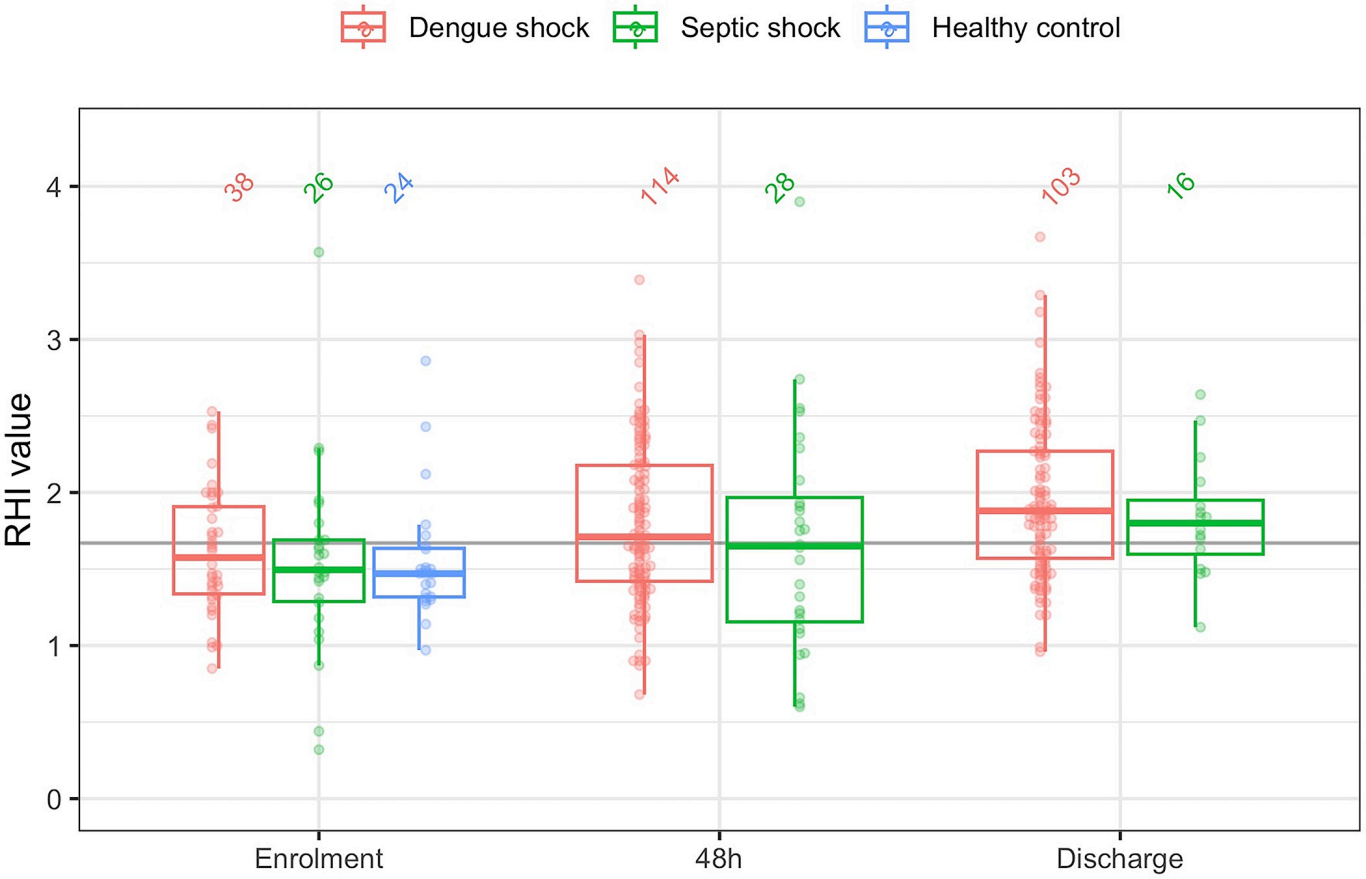
Serial Reactive hyperaemia index during hospital admission.

Fig 3 displays the RHI at enrolment, 48 hours later and hospital discharge. Due to the omission of EndoPAT in patients with platelet count <20x10^9^/L or DIC, RHI is only available for a subset of patients with DS at enrolment. In patients with DS and SS, median RHI was below the normal threshold (1.67) at enrolment, but increased above this threshold by 48 hours and hospital discharge. There was no significant difference in RHI between the patient groups. The RHI for healthy controls was unexpectedly low, with median RHI below both patient groups even at the time of enrolment. RHI was not associated with SOFA score, need for ICU admission or mortality (Tables 3 and 4).

**Table 3:** Association between SOFA score and biomarkers of inflammation, endothelial activation and endothelial glycocalyx breakdown at enrolment.

	N	Beta	95% CI^1^	p-value
RHI	38	-0.80	-2.5, 0.86	0.334
Ferritin (log2 ng/ml)	135	0.45	0.28, 0.62	**<0.001**
IL-6 (log2 pg/ml)	135	0.70	0.56, 0.85	**<0.001**
Angiopoietin-1 (log2 pg/ml)	135	0.12	-0.11, 0.36	0.309
Angiopoietin-2 (log2 pg/ml)	135	0.81	0.50, 1.1	**<0.001**
VCAM-1 (log2 pg/ml)	135	0.23	-0.34, 0.79	0.434
Hyaluronan (log2 ng/ml)	135	0.41	0.18, 0.65	**<0.001**
Endocan (log2 pg/ml)	135	0.92	0.53, 1.3	**<0.001**
ANP (log2 pg/ml)	135	0.47	0.10, 0.84	0.013
sTie-2 (log2 pg/ml)	135	-0.44	-0.85, -0.03	0.034
Heparan sulfate (log2 ug/ml)	135	0.22	0.06, 0.39	**0.009**
Syndecan-1 (log2 ng/ml)	135	0.53	0.35, 0.71	**<0.001**

^1^CI = Confidence Interval, RHI = reactive hyperaemia index, IL6 = interleukin-6, VCAM-1 = vascular cell adhesion molecule 1, ANP = atrial natriuretic peptide

**Table 4:** Association between need for ICU admission, mortality and biomarkers of inflammation, endothelial activation and endothelial glycocalyx breakdown at enrolment.

	Dengue shock				
	N	Event N	OR^1^	95% CI^1^	p-value
**Need for ICU admission**					
RHI	38	8	0.04	0.00, 0.47	0.028
Ferritin (log2 ng/ml)	135	34	1.55	1.21, 2.03	**<0.001**
IL-6 (log2 pg/ml)	135	34	2.61	1.79, 4.15	**<0.001**
Angiopoietin-1 (log2 pg/ml)	135	34	1.59	1.16, 2.24	**0.005**
Angiopoietin-2 (log2 pg/ml)	135	34	1.83	1.15, 3.05	0.014
VCAM-1 (log2 pg/ml)	135	34	0.52	0.25, 1.07	0.076
Hyaluronan (log2 ng/ml)	135	34	1.18	0.85, 1.71	0.353
Endocan (log2 pg/ml)	135	34	1.82	1.05, 3.30	0.038
ANP (log2 pg/ml)	135	34	2.36	1.43, 4.08	**0.001**
sTie-2 (log2 pg/ml)	135	34	0.80	0.47, 1.36	0.404
Heparan sulfate (log2 ug/ml)	135	34	1.12	0.89, 1.40	0.339
Syndecan-1 (log2 ng/ml)	135	34	2.02	1.47, 2.90	**<0.001**
**In-hospital mortality**					
RHI	38	2	0.04	0.00, 2.52	0.202
Ferritin (log2 ng/ml)	135	5	13.8	2.94, 284	**0.020**
IL-6 (log2 pg/ml)	135	5	4.49	2.14, 21.9	**0.005**
Angiopoietin-1 (log2 pg/ml)	135	5	3.62	1.60, 10.9	**0.006**
Angiopoietin-2 (log2 pg/ml)	135	5	8.06	2.55, 38.9	**0.002**
VCAM-1 (log2 pg/ml)	135	5	0.53	0.13, 2.68	0.390
Hyaluronan (log2 ng/ml)	135	5	1.34	0.65, 3.36	0.508
Endocan (log2 pg/ml)	135	5	3.36	1.02, 12.0	0.047
ANP (log2 pg/ml)	135	5	3.16	1.13, 9.74	0.031
sTie-2 (log2 pg/ml)	135	5	0.69	0.22, 2.35	0.537
Heparan sulfate (log2 ug/ml)	135	5	1.44	0.85, 2.68	0.201
Syndecan-1 (log2 ng/ml)	135	5	6.51	2.59, 26.0	**<0.001**

^1^OR = Odds Ratio, CI = Confidence Interval, RHI = reactive hyperaemia index, IL6 = interleukin-6, VCAM-1 = vascular cell adhesion molecule 1, ANP = atrial natriuretic peptide

### Biomarkers

**Fig 4:**
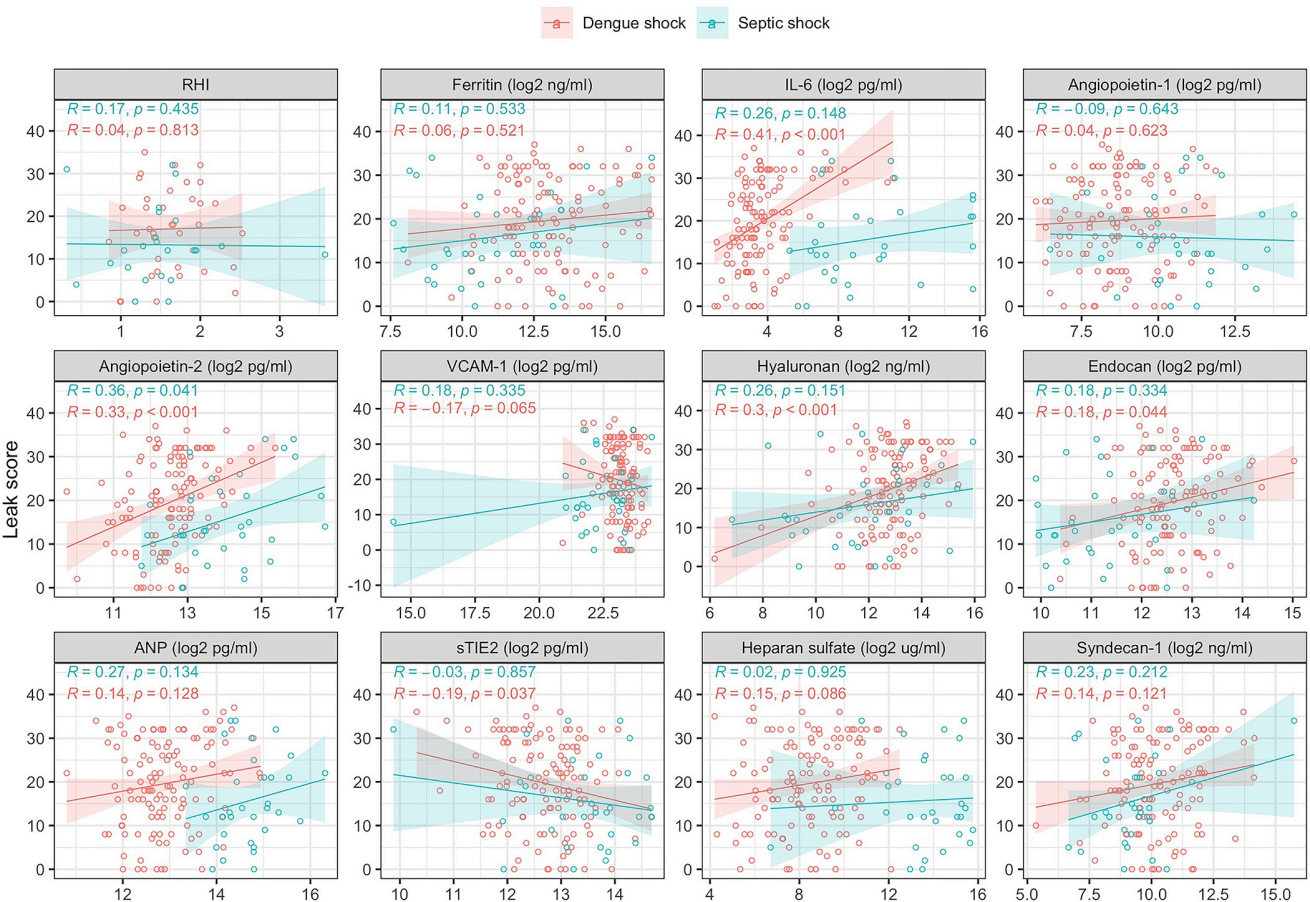
Correlation between pulmonary vascular leak score and biomarkers of inflammation, endothelial activation and glycocalyx breakdown at enrolment. RHI = reactive hyperaemia index, IL-6 = interleukin-6, VCAM-1 = vascular cell adhesion molecule 1, ANP = atrial natriuretic peptide.

**Fig 5:**
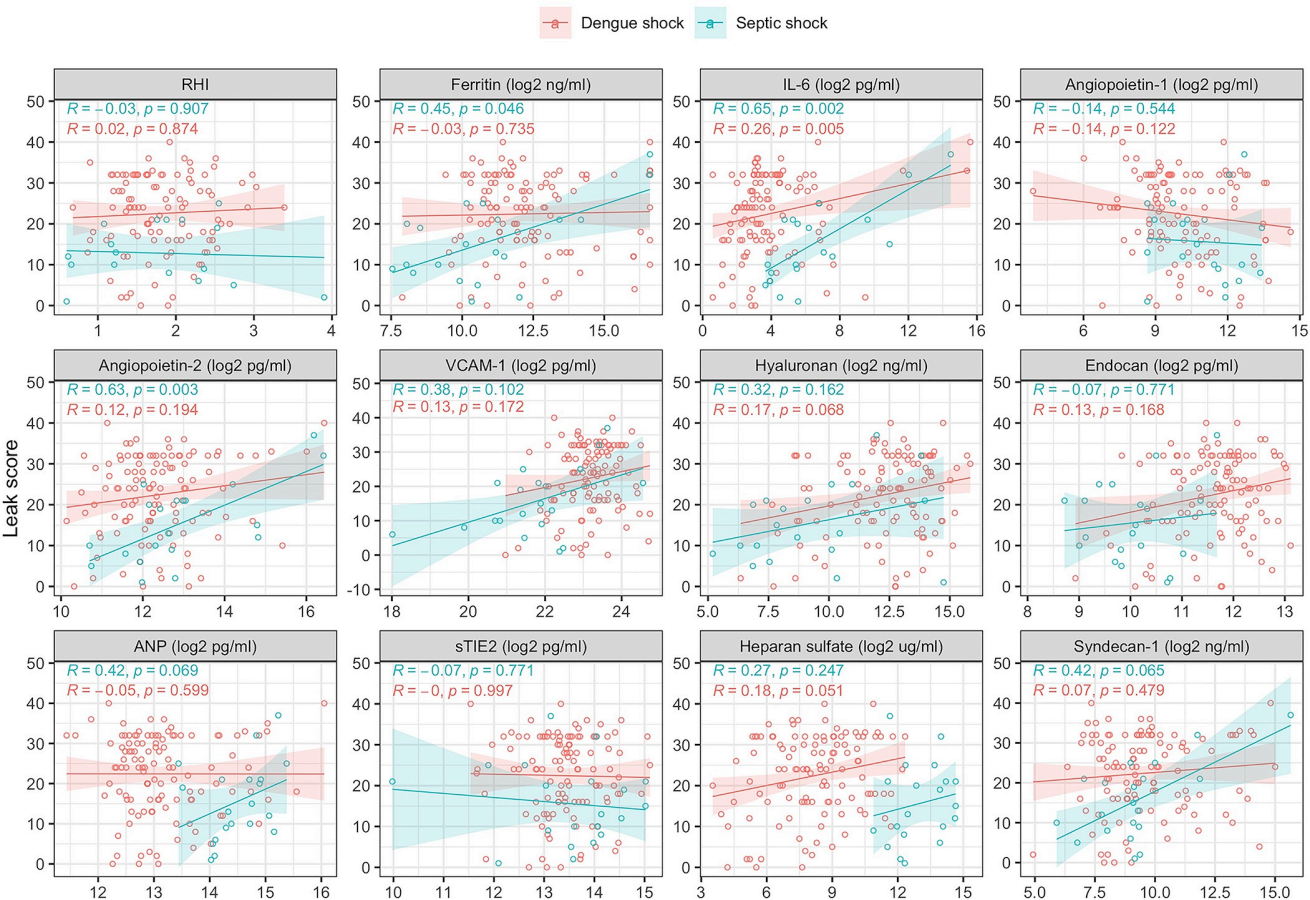
Correlation between pulmonary vascular leak score and biomarkers of inflammation, endothelial activation and glycocalyx breakdown at 48 hours after enrolment. RHI = reactive hyperaemia index, IL6 = interleukin-6, VCAM-1 = vascular cell adhesion molecule 1, ANP = atrial natriuretic peptide.

Table 5 and Fig C in S1 Text show the results of serial measurements of the following biomarkers: inflammatory (ferritin, IL-6), endothelial activation (Ang-1, Ang-2, sTie-2, VCAM-1), and glycocalyx degradation (hyaluronan, endocan, heparan sulfate and syndecan-1). Table 3 shows the association between biomarkers and SOFA score, and Table 4 shows the association between biomarkers and ICU admission and mortality for patients with dengue shock. Figs D, E, and F in S1 Text display these data graphically. Figs 4 and 5 show the correlation between biomarkers and the pulmonary vascular leak score at admission to hospital, and 48 hours later.

**Table 5:** Serial biomarkers of inflammation, endothelial activation and endothelial glycocalyx breakdown during hospital admission.

	n	Dengue shock (N = 135)^1^	n	Septic shock (N = 37)^1^	n	Healthy control (N = 25)^1^	Dengue shock vs Septic shock^2^	Dengue shock vs healthy control^2^	Septic shock vs healthy control^2^
Ferritin (ng/ml)									
Enrolment	135	7671 (3925;17251)	37	1645 (731; 6433)	25	68 (40; 121)	**p<0.001**	**p<0.001**	**p<0.001**
48 hours	131	4407 (2250; 10304)	31	1700 (965; 3830)			**p<0.001**	**p<0.001**	**p<0.001**
Discharge	108	1907 (1126; 3438)	18	837 (264; 1431)			**p<0.001**	**p<0.001**	**p<0.001**
IL-6 (pg/ml)									
Enrolment	135	9.9 (6.8; 17.4)	37	452.6 (140.7; 2898)	25	1.5 (1.5; 1.5)	**p<0.001**	**p<0.001**	**p<0.001**
48 hours	131	10.4 (6.7; 23.7)	31	63.0 (25.4; 491.5)			**p<0.001**	**p<0.001**	**p<0.001**
Discharge	109	3.3 (1.9; 6.7)	18	15.1 (6.9; 27.0)			**p<0.001**	**p<0.001**	**p<0.001**
Ang-1 (log2 pg/ml)									
Enrolment	135	8.7 (7.9; 9.6)	37	10.8 (9.9; 11.7)	25	12.0 (11.1;12.7)	**p<0.001**	**p<0.001**	**p = 0.004**
48 hours	131	10.0 (9.0; 11.6)	32	11.0 (10.1; 12.0)			p = 0.023	**p<0.001**	p = 0.016
Discharge	108	11.6 (10.8; 12.6)	17	11.8 (11.1; 12.6)			p = 0.739	p = 0.372	p = 0.800
Ang-2 (log2 pg/ml)									
Enrolment	135	12.6 (12.1; 13.0)	37	13.9 (13.1; 14.6)	25	10.6 (10.4; 11.3)	**p<0.001**	**p<0.001**	**p<0.001**
48 hours	131	12.3 (11.7; 13.0)	31	12.8 (12.0; 12.9)			p = 0.018	**p<0.001**	**p<0.001**
Discharge	108	11.7 (11.3; 12.1)	18	11.7 (11.1; 12.1)			p = 0.886	**p<0.001**	**p = 0.003**
sTie-2 (log2 pg/ml)									
Enrolment	135	12.8 (12.1; 13.1)	37	13.6 (13.0; 14.1)	25	12.2 (12.1; 12.8)	**p<0.001**	p = 0.046	**p<0.001**
48 hours	131	13.4 (13.0–13.9)	31	13.6 (13.1; 14.1)			p = 0.187	**p<0.001**	**p<0.001**
Discharge	108	14.2 (13.8; 14.6)	18	13.7 (13.5; 14.4)			p = 0.013	**p<0.001**	**p<0.001**
VCAM-1 (log2 pg/ml)									
Enrolment	135	23.2 (22.8; 23.5)	37	22.6 (21.7; 23.2)	25	19.0 (18.7; 19.2)	**p<0.001**	**p<0.001**	**p<0.001**
48 hours	131	23.2 (22.8; 23.6)	31	22.0 (20.8; 23.1)			**p<0.001**	**p<0.001**	**p<0.001**
Discharge	108	21.4 (20.9; 22.3)	18	20.2 (19.8; 21.3)			**p<0.001**	**p<0.001**	**p<0.001**
Hyaluronan (log2 ng/ml)									
Enrolment	135	12.9 (12.2; 13.4)	37	11.8 (10.0; 13.3)	25	4.5 (4.1; 5.4)	**p = 0.010**	**p<0.001**	**p<0.001**
48 hours	131	12.8 (11.5; 13.9)	31	9.6 (7.0; 11.0)			**p<0.001**	**p<0.001**	**p<0.001**
Discharge	108	7.4 (6.6; 8.8)	18	6.4 (4.5; 7.3)			**p = 0.006**	**p<0.001**	**p = 0.008**
Endocan (log2 pg/ml)									
Enrolment	135	12.6 (12.2; 13.1)	37	11.2 (10.6; 12.2)	25	9.1 (8.7; 9.3)	**p<0.001**	**p<0.001**	**p<0.001**
48 hours	131	11.8 (11.2; 12.2)	31	10.2 (9.4; 10.8)			**p<0.001**	**p<0.001**	**p<0.001**
Discharge	108	9.8 (9.3; 10.4)	18	9.7 (9.0; 10.8)			p = 0.775	**p<0.001**	p = 0.010
Heparan sulfate (log2 ug/ml)									
Enrolment	135	8.5 (7.3; 9.8)	37	14.0 (13.0; 15.1)	25	4.7 (2.8; 5.3)	**p<0.001**	**p<0.001**	**p<0.001**
48 hours	131	8.4 (7.0; 9.6)	31	13.3 (12.0; 14.1)			**p<0.001**	**p<0.001**	**p<0.001**
Discharge	108	7.5 (6.0; 8.6)	18	9.7 (7.8; 10.5)			**p<0.001**	**p<0.001**	**p<0.001**
Syndecan-1 (log2 ng/ml)									
Enrolment	135	10.1 (9.4; 11.3)	37	9.4 (8.9; 10.1)	25	5.2 (4.6; 5.5)	**p = 0.003**	**p<0.001**	**p<0.001**
48 hours	131	9.4 (8.3; 11.0)	31	9.2 (8.8; 10.0)			p = 0.573	**p<0.001**	**p<0.001**
Discharge	108	8.2 (7.2; 9.0)	18	8.9 (7.8; 9.0)			p = 0.347	**p<0.001**	**p<0.001**

^*1*^ *Median (25%; 75%)*

^*2*^*Wilcoxon rank sum test*. *Bold text indicates p<0*.*01*. *Abbreviations*: *IL6 = interleukin-6*, *Ang1 = Angiopoietin-1*, *Ang2 = angiopoietin-2*, *VCAM1 = vascular cell adhesion molecule 1*

### Biomarkers of inflammation

Although both ferritin and IL-6 were raised in patients with DS and SS, when compared to healthy controls, patients with DS had higher ferritin and lower IL-6 than those with SS.

Within the DS group, IL-6 and ferritin were associated with admission SOFA score (p<0.001 for both), ICU admission (p<0.001 for both) & mortality (IL-6: p = 0.005 & ferritin: p = 0.02); both biomarkers discriminated survivors and non-survivors at 48 hours and all patients who died from DS had pre-mortem ferritin ≥100,000ng/ml. Of all the biomarkers measured in this study, IL-6 was most strongly correlated with severity of pulmonary vascular leakage at study enrolment in patients with DS, p<0.001).

#### Biomarkers of endothelial activation

At enrolment, Ang-1 levels were lowest in patients with DS, though patients with SS also had lower Ang-1 than healthy controls. Ang-2 was increased in both shock groups compared to healthy controls, but levels were highest in patients with SS. VCAM-1 was raised in both groups with shock, but highest in those with DS.

For patients with DS, enrolment Ang-1 was associated with requirement for ICU admission (p = 0.005) & mortality (p = 0.006), but not with SOFA score. Ang-2 was associated with SOFA score (p<0.001) & mortality (p = 0.002), but not ICU admission. Although sTie-2 and VCAM-1 were elevated in patients with DS compared to healthy controls, there was no association between sTie-2 or VCAM-1 levels and SOFA score, ICU admission or mortality. Ang-2 was positively correlated with pulmonary vascular leak (R = 0.33, p<0.001) in patients with DS, but there was no significant correlation between Ang-1, sTie-2 or VCAM-1 and lung ultrasound scores.

#### Biomarkers of glycocalyx degradation

All glycocalyx biomarkers were higher in groups with shock versus healthy controls. Hyaluronan, endocan and SDC-1 levels were higher in patients with DS compared to SS, whereas heparan sulfate levels were highest in patients with SS.

Within the dengue shock group, although all of the glycocalyx biomarkers were positively associated with SOFA score at enrolment, only syndecan-1 was associated with ICU admission (p<0.001) and mortality (p<0.001). Hyaluronan was correlated with pulmonary vascular leak at enrolment (p<0.001).

## Discussion

The results of this study highlight several pathophysiological pathways in dengue shock which are associated with plasma leakage, and increased disease severity as measured by SOFA score, requirement for ICU admission and mortality. Until now, comparator groups in studies investigating biomarkers in dengue have been either healthy controls or patients with non-severe ‘other febrile illness’. As such, it hasn’t been clear whether the derangements seen in severe dengue are unique or simply features of the broad immune and endothelial dysregulation seen in other severe infections.

The results of this analysis indicate that while patients with both dengue shock and septic shock have evidence of pro-inflammatory hypercytokinaemia, there is a phenotypic difference between the inflammatory response in dengue shock (ferritin predominant) and septic shock (IL-6 predominant). Hyperferritinaemia has been reported as both a diagnostic and prognostic biomarker for dengue by several groups [12–15], but only one study has reported ferritin values quite as high as in our cohort; Kan et al found that mortality was twice as high in adults with severe dengue plus features of secondary haemophagocytic lymphohistiocytosis, compared to severe dengue alone [16]. Advancing this finding, we found that ferritin at enrolment was associated with mortality in a large group of adults with dengue shock, and that it was associated with other relevant clinical markers of disease severity such as SOFA score and requirement for ICU admission.

Although attempts to modulate the immunopathology in dengue with corticosteroids have been largely unsuccessful, this may be in part due to trials being conducted in an unselected patient population, most of whom had a high likelihood of good outcome [17,18]. COVID-19 cemented the theory that has emerged from many negative sepsis trials; that identifying and targeting the correct sub-population is key to both demonstrating clinical benefit and minimizing harm to patients through immune modulation. We have now begun recruiting to a phase II trial of the IL-1 receptor antagonist, Anakinra, in patients with moderate/severe dengue and ferritin>2000ng/ml. This will be the first trial of immunomodulation in dengue using biomarker-based enrichment for patient selection, and we hope that platforms to evaluate other host-directed therapeutics, such as the Janus kinase 1/2 inhibitor Baricitinib, will follow [19].

In this study, we also found that although IL-6 levels were lower in the group with dengue shock versus septic shock, IL-6 was markedly elevated at 48 hours in the few participants who did not survive their illness, and IL-6 was positively associated with pulmonary vascular leakage scores at both enrolment and 48 hours later. The literature on IL-6 in dengue is less robust than that for ferritin, but several groups have also found an association between elevated IL-6 and disease severity [20–24]. These results add to the body of evidence, and suggest that it may be appropriate to attempt IL-6 blockade with agents such as Tocilizumab in selected patient sub-groups. However, since IL-6 is less readily available at most hospital laboratories than the ferritin assay, it may be more complicated and costly to identify the group most likely to benefit from targeted therapy in a timely fashion. Future studies could explore whether the more readily measurable downstream inflammatory biomarker C-reactive protein might serve as a proxy indicator for patients with markedly deranged IL-6, and thus could help to identify a sub-population who may benefit from therapeutic IL-6 modulation.

With respect to the endothelial glycocalyx, we found that broad injury to the glycocalyx occurs in dengue shock, with elevated heparan sulfate, hyaluronan and SDC-1 in dengue shock versus healthy controls; however heparan sulfate levels were markedly higher in the septic shock group. To investigate this difference further, it would be helpful to measure the extent and timing of heparanase activity in both groups. Despite evidence that all components of the glycocalyx are shed during dengue shock, and elevated in association with SOFA score, only SDC-1 was associated with mortality. These results echo those found by us and others, that SDC-1 levels are consistently associated with disease severity in dengue [25,26]; our study extends these findings to report an association between SDC-1 levels and organ failure, requirement for ICU admission and death. One hypothesis to explain this observation may be that loss of the more superficial HS side chains and HA matrix has less severe functional consequences when compared to loss of the core proteoglycans such as SDC-1; indeed early loss of the sidechains may even render SDC-1 more susceptible to enzymatic cleavage by activated matrix metalloproteinases (MMP) [27]. On aggregate, the results suggest that strategies to reduce shedding of, or accelerate restitution of SDC-1 may be a viable approach for dengue shock. Reduction of SDC-1 shedding by MMP-9 inhibitors [28,29] or through sphingosine-1-phosphate mediated MMP suppression [30,31], or glycocalyx protective fluid strategies [32] are all potential therapeutic approaches to SDC-1 preservation. Indeed, although HS itself did not show clear associations with clinical outcomes in this study, inhibiting heparanase mediated shedding of HS side chains with heparin or Sulodexide are potential indirect strategies to protect SDC-1 from MMP mediated cleavage [33].

With respect to endothelial activation, Ang-1 was lower and Ang-2 higher in patients with DS versus healthy controls, indicating a loss of protective endothelial-stabilising and an excess of barrier disruptive signaling, together with shedding of the Tie2 receptor ectodomain. Several prior studies have reported associations between deranged Ang-1/2 and either plasma leakage or severe manifestations of dengue [34–38]; the positive correlation between Ang-2 levels and severity of pulmonary vascular leakage in our study adds to this evidence base. Furthermore, this is the first study to show an association between dysregulation of the Ang-Tie system and mortality in dengue as well as the first to report increased sTie-2 in dengue shock. Several experimental therapeutics targeting the Ang/Tie2 system are in development, or early clinical trials. Tie-2 receptor antagonists including Vasculotide and AV-001 (Vasomune therapeutics) have shown promise in animal models of sepsis and influenza [39,40]; a phase 2a study of AV-001 in adults with pneumonia is underway [41], but others including the Ang-2 binding and Tie-2 activating antibody (ABTAA) have also shown promise in preclinical models of sepsis [42].

VCAM-1 was markedly elevated during dengue shock, but was not associated with pulmonary vascular leakage, SOFA score, ICU admission or mortality. The findings of this study do not support prioritization of VCAM-1 as a therapeutic target; in any case, there are no promising pharmacological candidates to target VCAM-1 mediated endothelial activation at present.

This study presents a large and carefully characterized cohort of adults with dengue and septic shock. This is one of the few prospective studies in which there are severe adverse outcomes reported, including requirements for organ support and mortality. However, low event numbers limit statistical power to determine associations between biomarkers and these adverse outcomes. Other limitations included the use of a novel scoring method for pulmonary vascular leakage; although it was considered important to adopt the more widely used pulmonary ARDS scoring systems to account for the profound plasma leakage that occurs in dengue shock, the adapted score has not been formally validated, and this should be undertaken before more widespread adoption. The decision to measure biomarkers reflecting a range of pathophysiological processes necessarily limited the number of biomarkers that could be measured for each; in particular, measuring only two biomarkers of inflammation limits the capacity to interpret the complex interplay between inflammatory pathways underlying the end point of shock.

### Summary and conclusions

This study outlines several new associations between biomarkers of inflammation, glycocalyx breakdown and endothelial activation, and clinically relevant outcomes in dengue shock. The striking association between biomarkers of inflammation and clinical outcomes suggests that host-directed immunomodulation should be explored in future therapeutic trials. With the addition of the Angiopoietin/Tie2 results to the literature, there is accumulating evidence to support candidate therapeutics targeting this pathway to move into clinical trials for dengue. With respect to development of novel therapeutics targeting the endothelial glycocalyx, those aimed at reducing SDC-1 breakdown should be a priority, though the complex interplay between the effectors of endothelial activation and sheddases may render therapeutics targeting other linked pathophysiological processes, including Ang-2 and heparanase, also indirect candidates for SDC-1 preservation. To complement studies such as this, organ on chip systems may facilitate screening of compounds targeting intracellular pathways involved in endothelial permeability [43].

## Supporting information

S1 Text Fig A. Daily vital signs for patients with dengue shock and septic shock. Fig B. Routine haematology and biochemistry tests for patients with dengue shock and septic shock. Fig C. Serial results of biomarkers of inflammation (top row), endothelial activation (middle row) and endothelial glycocalyx breakdown (bottom row) measured at enrolment, 48 hours later, and at hospital discharge. Fig D. Association between SOFA score and biomarkers of inflammation, endothelial activation and glycocalyx breakdown at enrolment. Fig E. Biomarkers of inflammation, endothelial activation and endothelial glycocalyx breakdown, sub-divided by requirement for ICU admission. Fig F. Biomarkers of inflammation, endothelial activation and endothelial glycocalyx breakdown, sub-divided by mortality.
(DOCX)
