# Supplementary material for: Endothelial and inflammatory pathophysiology in dengue shock: New insights from a prospective cohort study in Vietnam
Source: PLoS Negl Trop Dis. 2024 Mar 27;18(3):e0012071. doi: 10.1371/journal.pntd.0012071 (PMC11020502; doi:10.1371/journal.pntd.0012071)
Supplement: S1 Text — Fig A. Daily vital signs for patients with dengue shock and septic shock. Fig B. Routine haematology and biochemistry tests for patients with dengue shock and septic shock. Fig C. Serial results of biomarkers of inflammation (top row), endothelial activation (middle row) and endothelial glycocalyx breakdown (bottom row) measured at enrolment, 48 hours later, and at hospital discharge. Fig D. Association between SOFA score and biomarkers of inflammation, endothelial activation and glycocalyx breakdown at enrolment. Fig E. Biomarkers of inflammation, endothelial activation and endothelial glycocalyx breakdown, sub-divided by requirement for ICU admission. Fig F. Biomarkers of inflammation, endothelial activation and endothelial glycocalyx breakdown, sub-divided by mortality. (DOCX) [file pntd.0012071.s001.docx]

S1 Text: Fig A. Daily vital signs for patients with dengue shock and septic shock


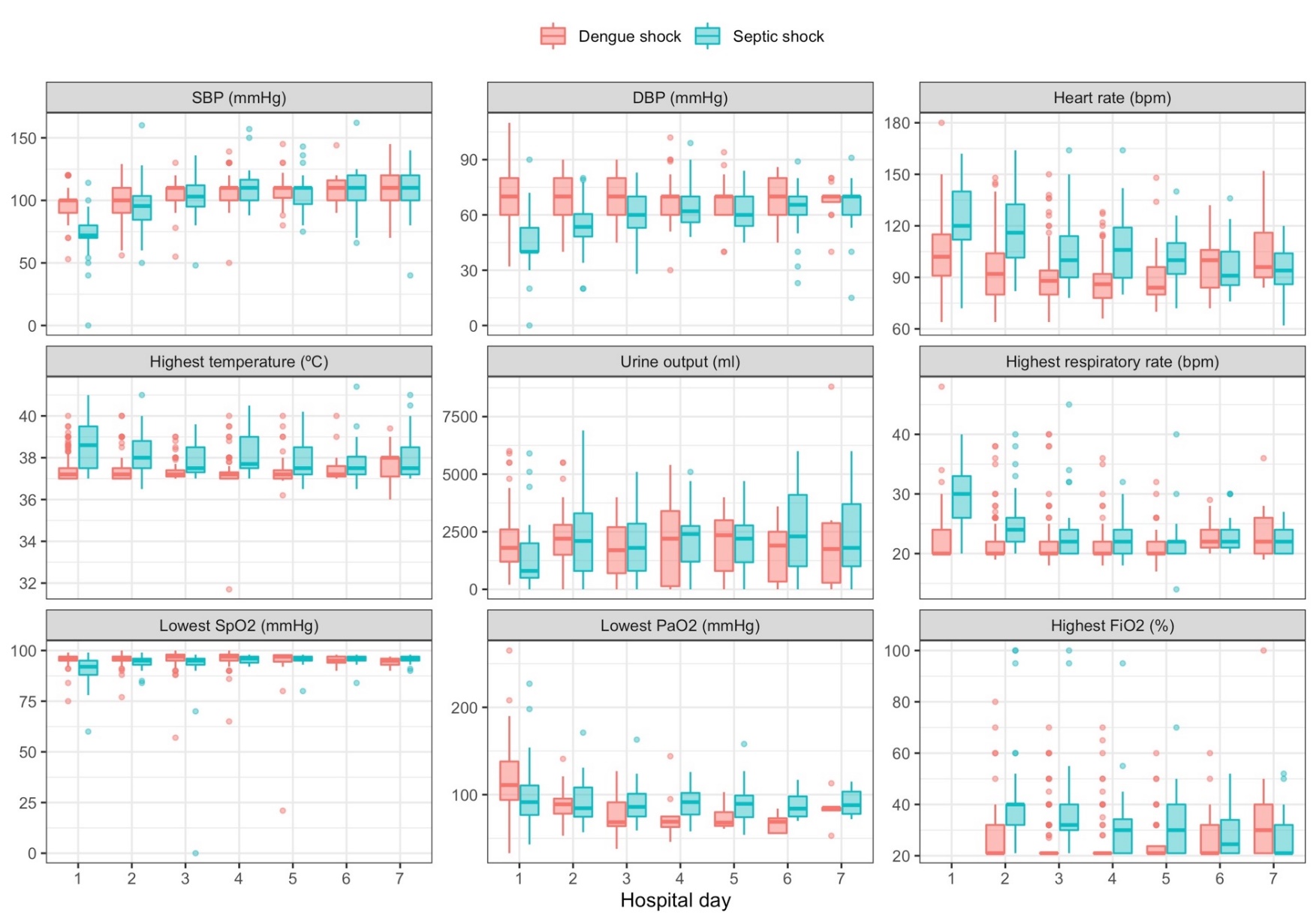


SBP=systolic blood pressure, DBP=diastolic blood pressure

S1 Text: Fig B. Routine haematology and biochemistry tests for patients with dengue shock and septic shock


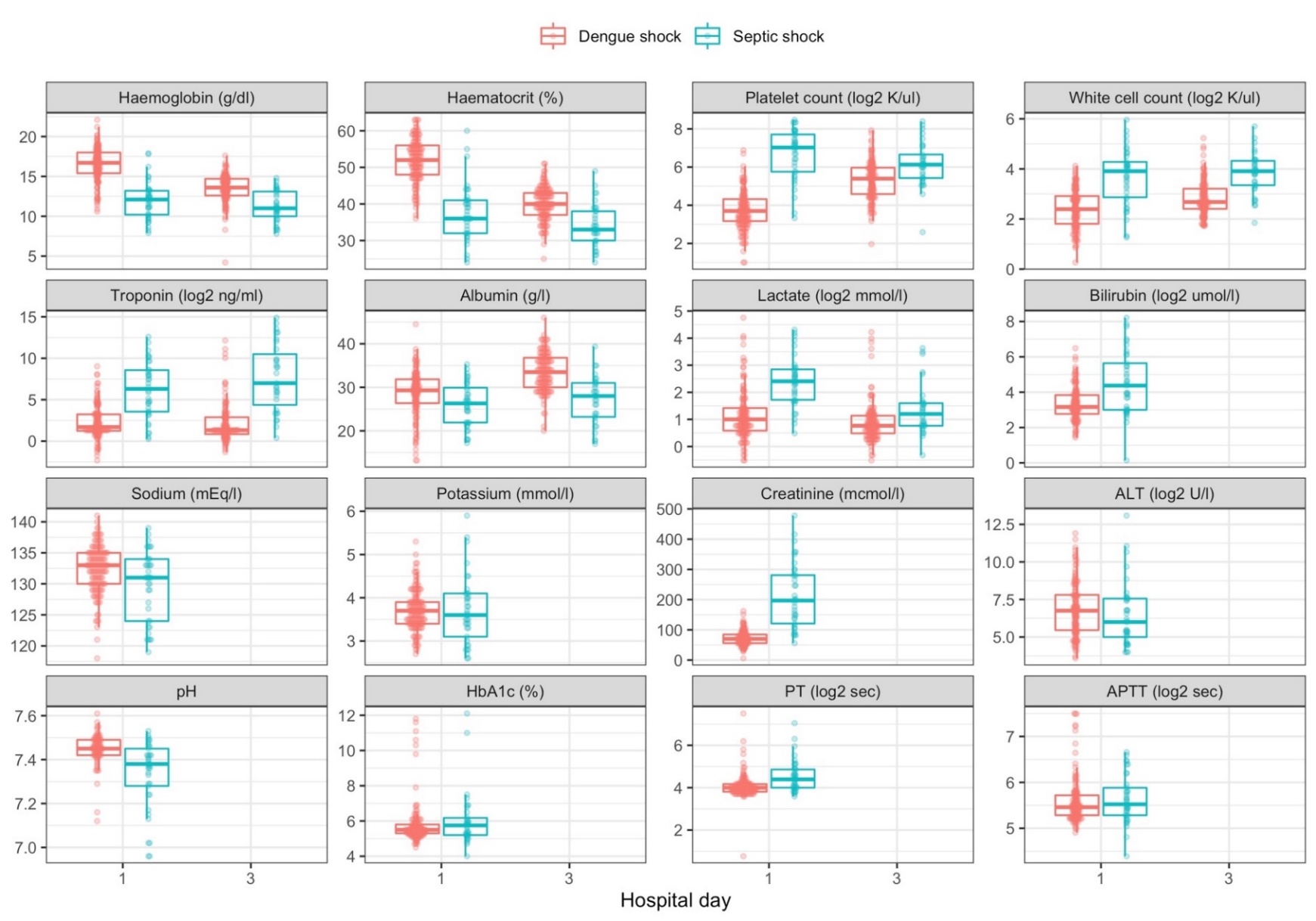


*Hospital day 1 = enrolment, hospital day 3 = 48 hours later.*

S1 Text: Fig C. Serial results of biomarkers of inflammation (top row), endothelial activation (middle row) and endothelial glycocalyx breakdown (bottom row) measured at enrolment, 48 hours later, and at hospital discharge.


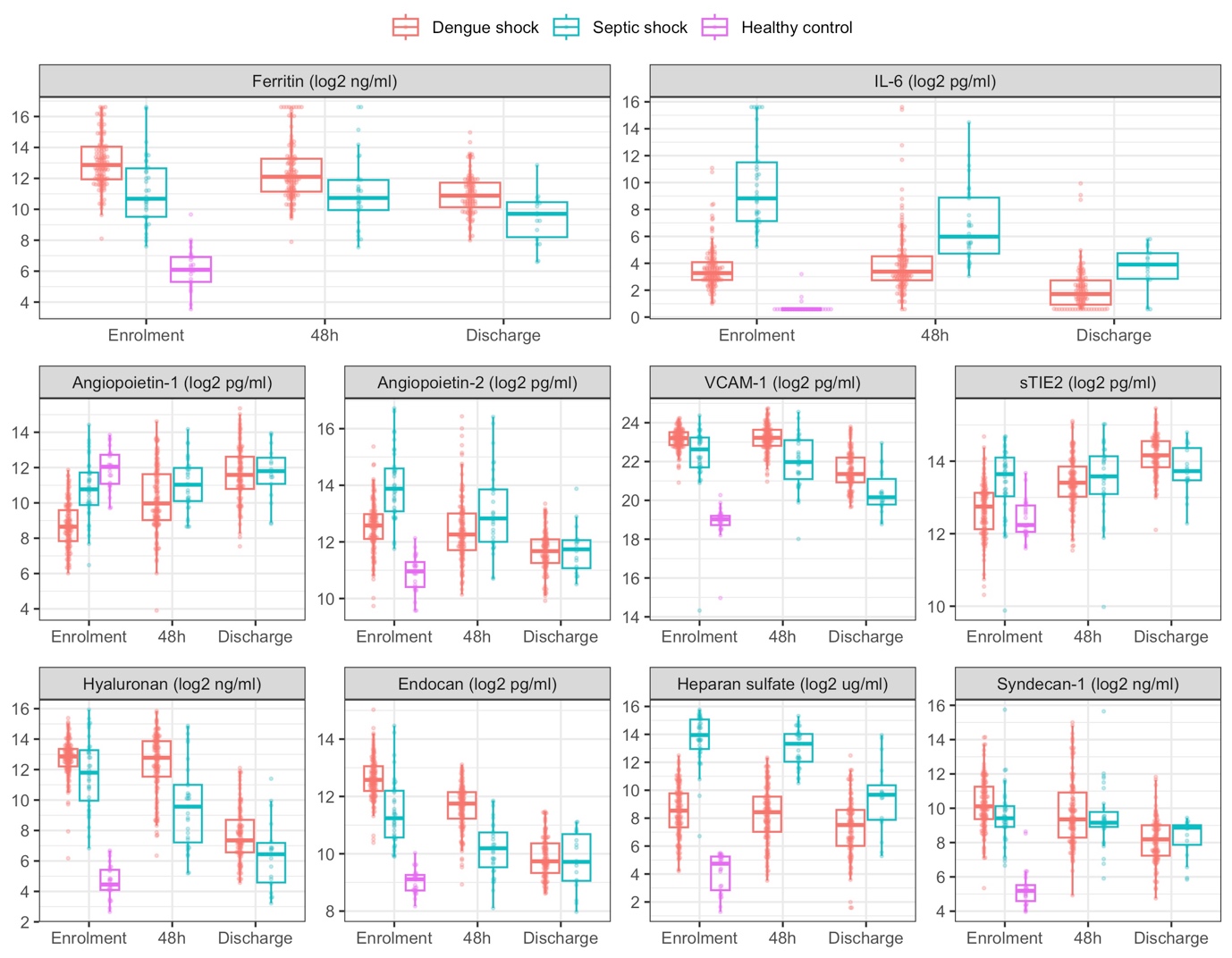


S1 Text: Fig D. Association between SOFA score and biomarkers of inflammation, endothelial activation and glycocalyx breakdown at enrolment


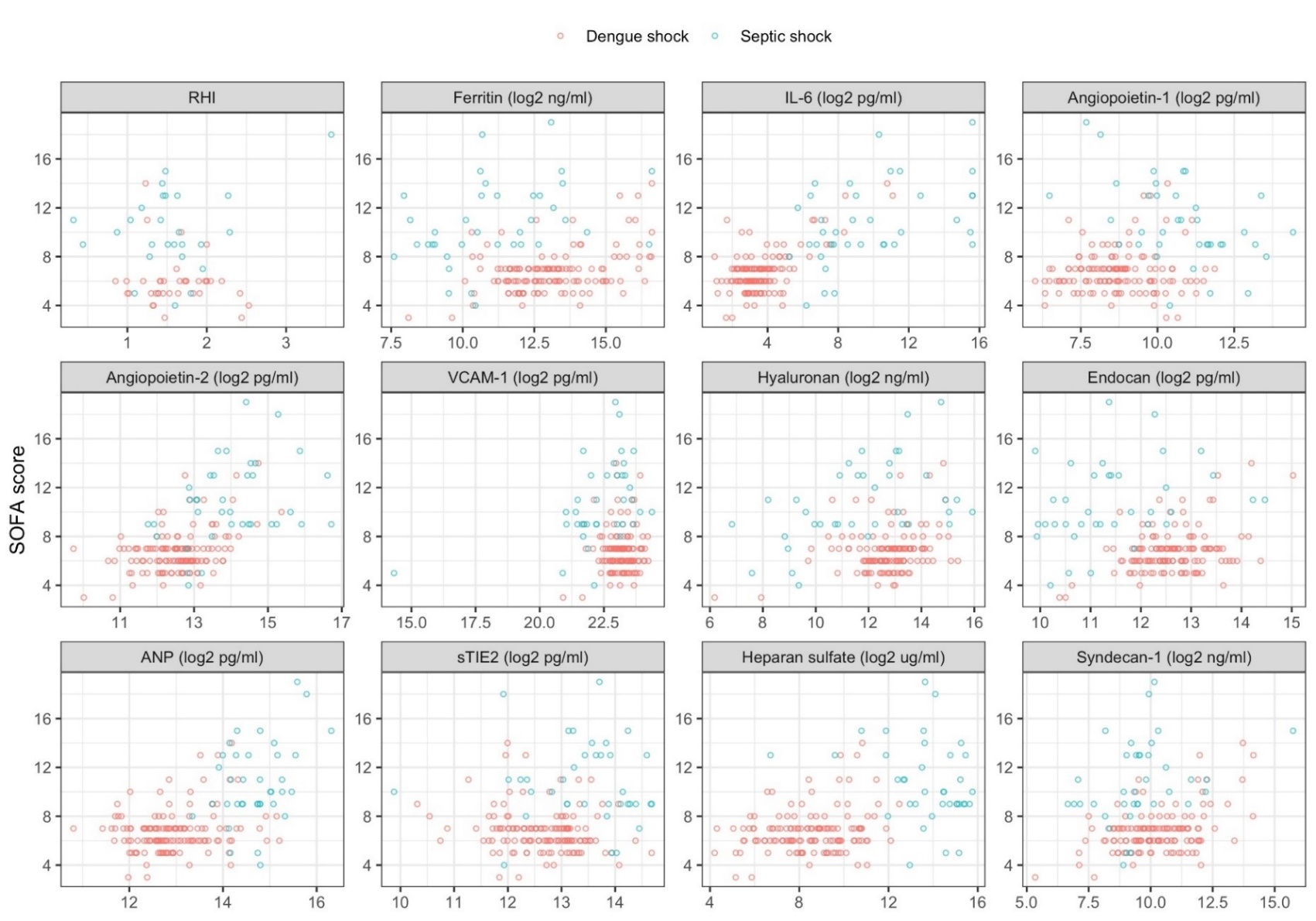


RHI = reactive hyperaemia index, IL6 = interleukin-6, VCAM-1 = vascular cell adhesion molecule 1, ANP = atrial natriuretic peptide

S1 Text: Fig E. Biomarkers of inflammation, endothelial activation and endothelial glycocalyx breakdown sub-divided by requirement for ICU admission


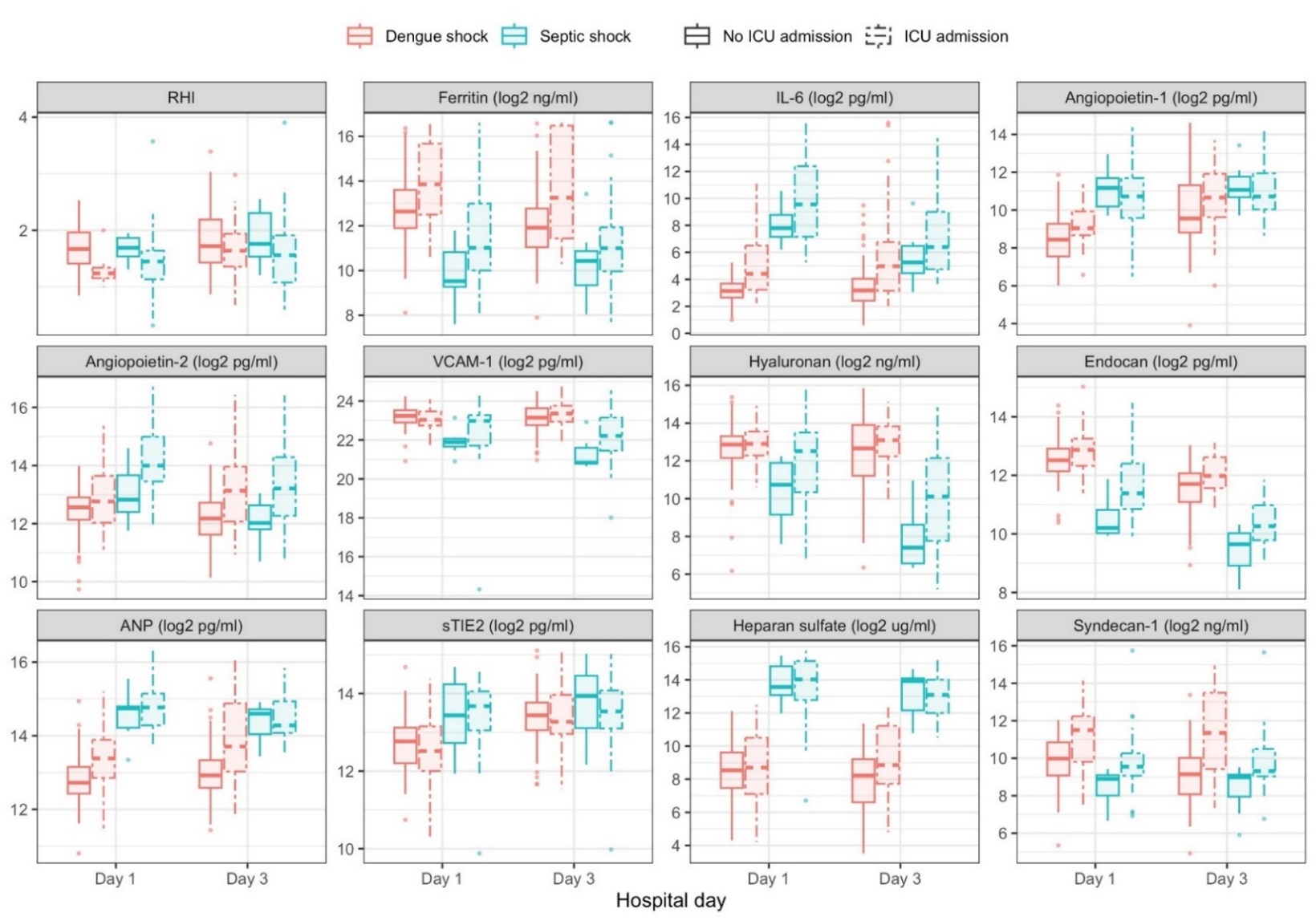


Hospital day 1=enrolment, day 3=48 hours later. RHI = reactive hyperaemia index, IL6 = interleukin-6, VCAM-1 = vascular cell adhesion molecule 1, ANP = atrial natriuretic peptide

S1 Text: Fig F. Biomarkers of inflammation, endothelial activation and endothelial glycocalyx breakdown sub-divided by mortality


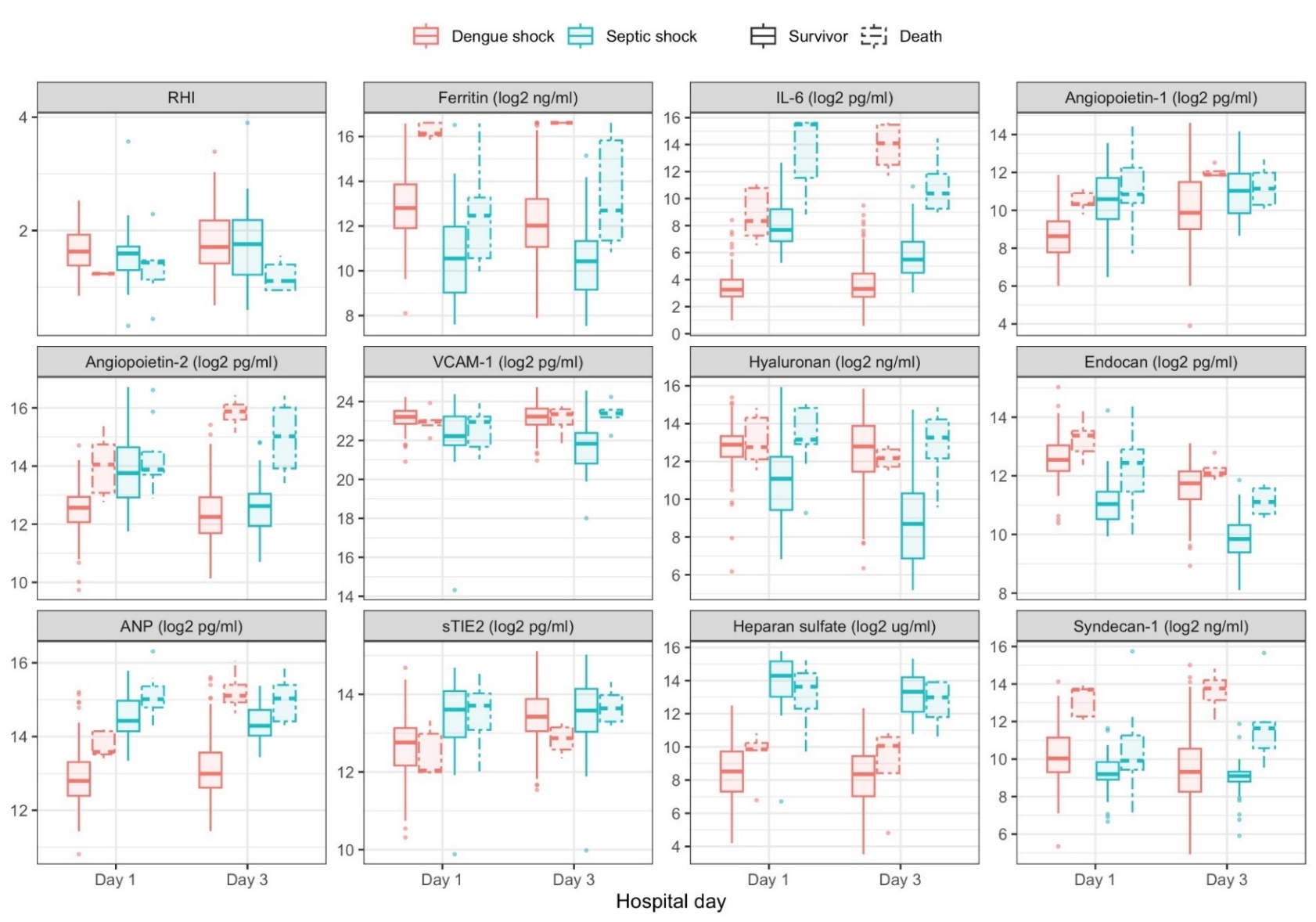


Hospital day 1=enrolment, day 3=48 hours later. RHI = reactive hyperaemia index, IL6 = interleukin-6, VCAM-1 = vascular cell adhesion molecule 1, ANP = atrial natriuretic peptide
